# Antibiotic prescribing knowledge: A brief survey of providers and staff at an ambulatory cancer center during Antibiotic Awareness Week 2019

**DOI:** 10.1017/ash.2021.218

**Published:** 2022-02-04

**Authors:** Elizabeth A. Gulleen, Elizabeth M. Krantz, Jacqlynn Zier, Pooja Bhattacharyya, Olivia Kates, Lisa So, Ania Sweet, Leah H. Yoke, Steven A. Pergam, Catherine Liu

**Affiliations:** 1 Vaccine and Infectious Diseases Division, Fred Hutchinson Cancer Research Center, Seattle, Washington; 2 Allergy and Infectious Diseases Division, Department of Medicine, University of Washington, Seattle, Washington; 3 Eastern Washington University, Spokane, Washington; 4 Department of Oncology, Department of Medicine, University of Washington, Seattle, Washington; 5 Division of Infectious Diseases, Department of Medicine, Johns Hopkins Medicine, Baltimore, Maryland; 6 Seattle Cancer Care Alliance, Seattle, Washington

## Abstract

We surveyed healthcare professionals at a cancer center regarding their knowledge and perceptions of antibiotic use. Most knew the term “antimicrobial stewardship.” Nurses and other staff were less likely than pharmacists or providers to answer knowledge-based questions correctly. Opportunities exist to improve antibiotic knowledge among cancer center staff.

Patients with cancer are at high risk of developing infections that require antibiotic therapy.^1^ Although antibiotics can be lifesaving, inappropriate antibiotic use is associated with increased antimicrobial resistance and adverse patient outcomes.^2^ We previously found that >30% of patients who were diagnosed with upper respiratory infections (URIs) at our ambulatory cancer center received antibiotics, even though most URIs are caused by viruses.^3^ Most antimicrobial stewardship interventions target pharmacists and prescribers. However, recognition that successful stewardship requires multiprofessional engagement is growing.^4^ In 2017, we established an antimicrobial stewardship program at our ambulatory cancer center. We conducted a survey to assess antibiotic knowledge and perceptions among healthcare professionals (HCPs) at our center.

## Methods

We invited all staff who attended an Antibiotic Awareness Week event at the Seattle Cancer Care Alliance on November 15, 2019, to complete a self-administered paper survey at the event (Table 1). During the event, members of our antimicrobial stewardship team were on site to answer questions, and we displayed posters showcasing our program’s efforts. We distributed the answer key via e-mail as our main educational intervention 2 weeks after the event. At 7 weeks after the event, we tested knowledge retention by inviting staff to answer the same questions in an electronic postevent survey. This study was approved by the Fred Hutch Institutional Review Board.

**Table 1.** Participant Responses to the 2019 Seattle Cancer Care Alliance Antibiotic Awareness Week Event Survey by Employee Role^a^

Survey Questions	All	Providers^c^	Pharmacists	Nurses	Other^d^	*P* Value^e^
No. of respondents, no. (%)^b^	159 (100)	30 (19)	22 (14)	32 (20)	75 (47)	…
**Q1. Are you aware of the term “antibiotic stewardship”?**						
I know a lot about it and support its practice	62 (39)	14 (47)	19 (86)	11 (34)	18 (24)	…
I have heard of it and know a little about it	58 (36)	13 (43)	3 (14)	14 (44)	28 (37)	…
I have never heard about it and do not know what it means	25 (16)	0 (0)	0 (0)	5 (16)	20 (27)	…
No response	14 (9)	3 (10)	0 (0)	2 (6)	9 (12)	…
**Q2 You can prevent the spread of antibiotic resistant bacteria by cleaning your hands.**						
True	142 (89)	28 (93)	21 (95)	28 (88)	65 (87)	.10
**Q3. A potentially serious side effect of antibiotics includes** ***C. difficile*** **infection.**						
True	143 (90)	30 (100)	22 (100)	29 (91)	62 (83)	.001
**Q4. Most people who believe they are allergic to penicillin can take penicillin without a problem.**						
True	100 (63)	30 (100)	22 (100)	23 (72)	25 (33)	<.001
**Q5. Many upper respiratory tract infections are caused by bacteria and should be treated with antibiotics**						
False	121 (76)	30 (100)	22 (100)	26 (81)	43 (57)	<.001
**Q6. Your cold will not last as long if you take antibiotics.**						
False	147 (92)	30 (100)	22 (100)	32 (100)	63 (84)	<.001
**Q7. When sputum or mucus turns green, antibiotics should be prescribed.**						
False	112 (70)	29 (97)	18 (82)	30 (94)	35 (47)	<.001
**Q8. If a patient with neutropenia (low white blood cell count) has a cough and runny nose, they should always take antibiotics due to their weakened immune system.**						
False	120 (75)	28 (93)	16 (73)	25 (78)	51 (68)	.007
**Q9. For which of the following illnesses (strep throat, diarrhea, influenza, common cold, runny nose) are antibiotics typically needed?** ^f^						
Strep throat	122 (77)	27 (90)	19 (86)	27 (84)	49 (65)	.001

a For questions 2-9, only the correct responses are listed. Missingness for these questions ranged from 1% to 5% and was counted as incorrect.

b Of the 161 respondents, 4 did not report employee role and were excluded from analysis.

c Providers included doctors, physician assistants, and nurse practitioners.

d Other staff included nursing assistants, administrators, licensed practical nurses, MRI technologists, PhD students, respiratory therapists, clinical research assistants, couriers, imaging technologists, medical students, nuclear medicine technologists, phlebotomists, program assistants, research managers, respiratory therapists, technical editors, and technologists.

e Based on 1-sided Cochran-Armitage test for trend by healthcare worker role.

f Participants could select 1 or more responses. Only those who selected strep throat and no other responses were considered to correctly answer the question.

### Statistical analysis

To compare antibiotic knowledge by staff role, we excluded respondents who did not indicate their clinical role from analysis. For knowledge-based questions, we assigned each correct true- or-false or multiple-choice question a value of 1; we considered unanswered questions to be incorrect. For each participant, we added the number of correct answers to create a cumulative score ranging from 0 to 8.

We analyzed survey responses using nonparametric tests. We used the Jonckheere-Terpstra test to test for decreasing trend in scores by staff role, the Mann-Whitney test to compare scores between 2 groups, a 1-sided Cochran-Armitage test to test for decreasing trend in correct responses for individual survey questions, and a Wilcoxon signed-rank test to compare paired event and postevent survey scores. We used SAS version 9.4 software (SAS Institute, Cary, NC) for all analyses. We considered a *P* value <.05 statistically significant.

## Results

### Participant characteristics

Among 163 individuals who completed the event survey, 159 indicated their staff role and were included in the analysis: 30 providers (19%), 22 pharmacists (14%), 32 nurses (20%), and 75 other staff (47%) (Supplementary Table 1).

### Event survey results

Of 159 respondents, 120 (75%) had some awareness of the term “antimicrobial stewardship” (Table 1). Among the 25 respondents who were unfamiliar with the term, 20 (80%) were other staff and 5 (20%) were nurses.

**Fig. 1.**
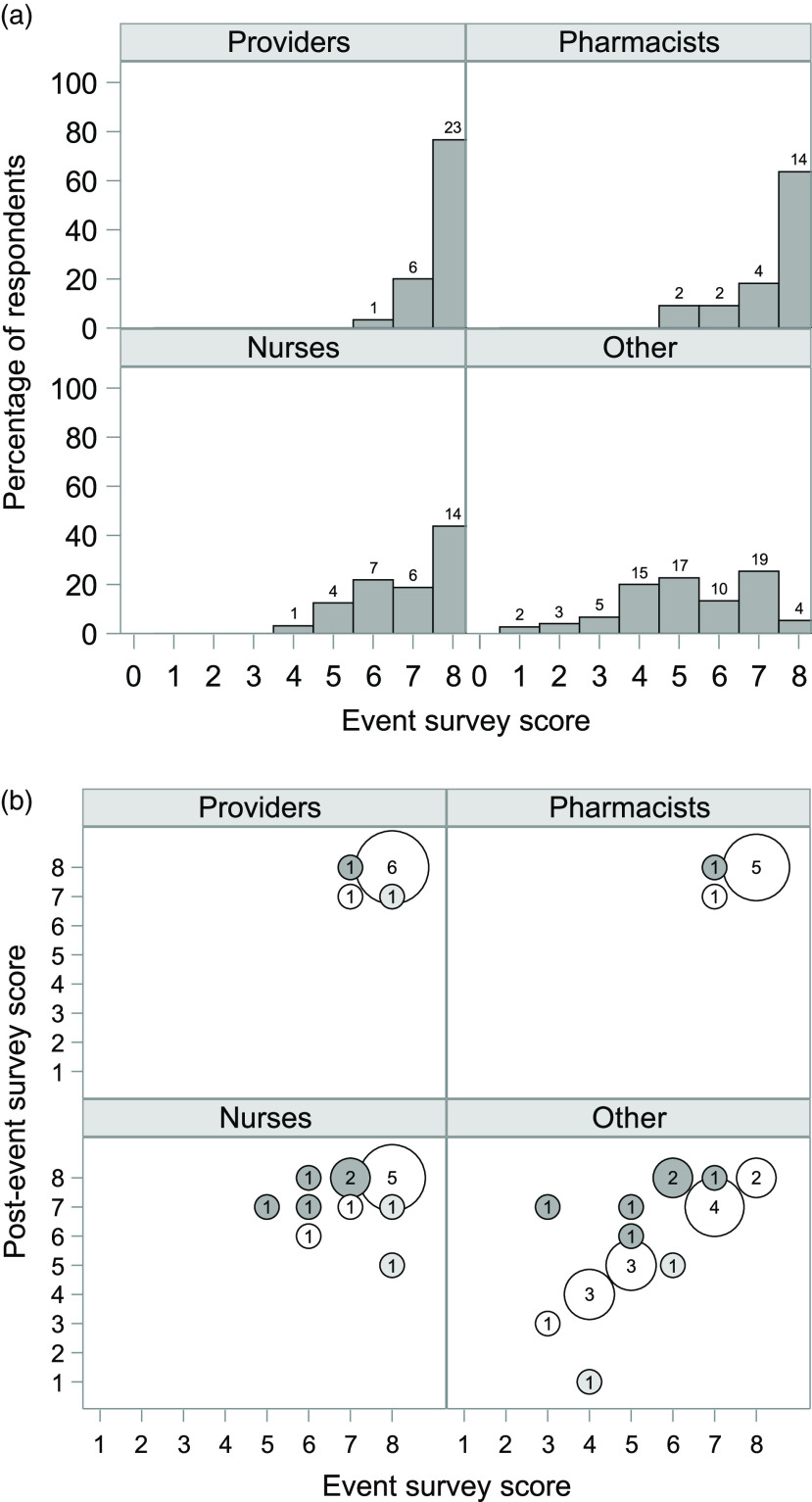
Data showing the number of correctly answered questions by respondent role.^a^ (a) The number of questions correctly answered at the event survey (event survey score) (n = 159). Numbers above the bars show the number of respondents with each score. The number of correct answers decreased from providers to pharmacists to nurses to other staff (*P* < .001 for trend, Jonckheere-Terpstra test). (b) Paired data for number of questions correctly answered at event (event survey score) and postevent survey (postevent survey score; N = 51). Size of the bubble corresponds to the number of participants in each category, which is shown in the center of each bubble. Light grey bubbles correspond to scores that decreased from the event to after the event; dark grey bubbles correspond to scores that increased; and unfilled bubbles correspond to scores that remained the same. We detected no significant difference between the event and postevent survey scores (Wilcoxon signed-rank test, *P* = 0.11). ^a^ Providers included doctors, physician assistants, and nurse practitioners. Other staff included nursing assistants, administrators, licensed practical nurses, MRI technologists, PhD students, respiratory therapists, clinical research assistants, couriers, imaging technologists, medical students, nuclear medicine technologists, phlebotomists, program assistants, research managers, respiratory therapists, technical editors, and technologists.

The median event survey score was 7 (interquartile range, 5–8). Scores decreased from providers to pharmacists to nurses to other staff (*P* < .001 for trend) (Fig. 1A). The 120 respondents who were familiar with the term “antimicrobial stewardship” scored higher on the event survey than the 25 who were unfamiliar with the term (median 7.0 vs 5.0; *P* < .001).

Table 1 shows the number of respondents who correctly answered each question by staff role. Of 159 respondents, 142 (89%) knew that hand hygiene prevents the spread of resistant bacteria; this did not differ by staff role (*P* = .10 for trend).

Regarding respiratory tract infections, all healthcare providers and pharmacists, 26 (81%) of 32 nurses, and 43 (57%) of 75 other staff correctly identified this statement as false: “many upper respiratory tract infections are caused by bacteria and should be treated with antibiotics” (*P* < .001 for trend). Although most providers, pharmacists, and nurses knew that antibiotics are not needed when sputum turns green, only 35 (47%) of 75 other staff knew this (*P* < .001 for trend). Also, 2 (7%) of 30 healthcare providers, 6 (27%) of 22 pharmacists, 6 (19%) of 32 nurses, and 21 (28%) of 75 other staff incorrectly thought that a patient with neutropenia who develops a cough and runny nose should always take antibiotics (*P* = .007 for trend). All providers, pharmacists, and nurses, as well as 63 (84%) of 75 other staff knew that antibiotics do not shorten the duration of a cold (*P* < .001 for trend).

When asked to identify which of several common illnesses typically require antibiotic therapy, 27 (90%) of 30 healthcare providers, 19 (86%) of 22 pharmacists, 27 (84%) of 32 nurses, and 49 (65%) of 75 other staff knew that only strep throat necessitates antibiotics (*P* = .001 for trend). Nurses and other staff were less likely to know that most people who believe they are allergic to penicillin can take penicillin without issue (*P* < .001 for trend).

### Postevent survey results

Of 159 respondents, 51 (32%) completed the postevent survey (Supplementary Table 1). Of the 9 respondents who initially reported they had never heard the term “antimicrobial stewardship,” 7 reported familiarity with the term in the postevent survey (Supplementary Fig. 2). There was no significant difference between the event and postevent survey scores (*P* = .11) (Fig. 1B).

## Discussion

Our survey results have highlighted differences in knowledge of appropriate antimicrobial use among staff working at a cancer center. Healthcare providers and pharmacists demonstrated greater knowledge than nurses or other staff. Those who had some familiarity with the term “antimicrobial stewardship” scored higher on the event survey than those who were unfamiliar with the term.

Traditionally, antimicrobial stewardship interventions target providers and pharmacists. However, stewardship is more successful when practiced within the multidisciplinary healthcare team.^4,5^ It is important to include nurses on the stewardship team because they are responsible for stewardship-related activities such as patient education and antibiotic administration.^4–6^ We found that most nurses had some familiarity with antimicrobial stewardship. However, >1 in 4 were unaware that most patients who reported a penicillin allergy could safely take a penicillin. Because nurses are responsible for medication reconciliation and allergy documentation, this represents an opportunity for targeted education.

Almost 90% of respondents recognized the importance of hand hygiene in preventing the spread of antimicrobial resistance. This rate did not differ by staff role, likely reflecting our ongoing hand hygiene efforts that target all HCPs. Conversely, nearly 30% of other staff were unfamiliar with the term antimicrobial stewardship and more than half were unaware that antibiotics had limited utility in managing URIs. Because cancer care is multidisciplinary and longitudinal, patients encounter numerous HCPs over the course of treatment. Based on the successful model of hand hygiene education, teaching all members of the multidisciplinary healthcare team about the importance of antimicrobial stewardship can create an environment that supports appropriate antibiotic use and sets reasonable patient expectations.^7^ Ensuring that patient-facing HCPs understand the limited utility of antibiotics for treating URIs could also improve patient knowledge.

Our study had several limitations. Only those who attended the Antibiotic Awareness Week event completed the survey, which introduced a response bias. Because the survey was completed at a single cancer care center, results may not be generalizable. The low response rate in our postevent survey may also limit generalizability regarding knowledge retention. Furthermore, our intervention consisted of distributing educational material (ie, survey answer key), which was insufficient to ensure knowledge retention.^8,9^ However, we will use the survey results to develop educational interventions targeting nonprovider, nonpharmacist staff.

Opportunities exist to improve antimicrobial stewardship education across the spectrum of HCPs at our cancer center, specifically among nurses and other staff. Further work is needed to determine the optimal approach to staff outreach and education.

## Supplementary material

For supplementary material accompanying this paper visit https://doi.org/10.1017/ash.2021.218. click here to view supplementary material
