# Supplementary material for: Antibiotic prescribing knowledge: A brief survey of providers and staff at an ambulatory cancer center during Antibiotic Awareness Week 2019
Source: Antimicrob Steward Healthc Epidemiol. 2022 Feb 4;2(1):e18. doi: 10.1017/ash.2021.218 (PMC9614796; doi:10.1017/ash.2021.218)
Supplement: Supplementary file 1 [file S2732494X21002187sup001.docx]

| **Supplemental Table 1.** Baseline demographics of Fred Hutchinson Cancer Research Center/Seattle Cancer Care Alliance staff who responded to the 2019 Antimicrobial Awareness Week survey and were eligible for analysis.^a^ | | | |
| --- | --- | --- | --- |
| **Characteristics** | **Completed Only Event Survey** | **Completed Event and Post-event Survey** | **All Participants who Completed Event Survey** |
| **Number of Respondents, n (%)** | 108 (68) | 51 (32) | 159 |
| **Employee Role Category, n (%)** |  |  |  |
| Providers^b^ | 21 (19) | 9 (18) | 30 (19) |
| Pharmacists | 15 (14) | 7 (14) | 22 (14) |
| Nurses | 18 (17) | 14 (27) | 32 (20) |
| Other^c^ | 54 (50) | 21 (41) | 75 (47) |
| **Years in Role, n (%)** |  |  |  |
| 0-4 | 53 (49) | 25 (49) | 78 (49) |
| 5-9 | 25 (23) | 9 (18) | 34 (21) |
| 10-14 | 12 (11) | 7 (14) | 19 (12) |
| 15+ | 15 (14) | 8 (16) | 23 (14) |
| No response | 3 (3) | 2 (4) | 5 (3) |
| **Service, n (%)** |  |  |  |
| Transplant | 19 (18) | 15 (29) | 34 (21) |
| Non-transplant | 59 (55) | 29 (57) | 88 (55) |
| No response | 30 (28) | 7 (14) | 37 (23) |
| ^a^ ­­­­Of the 161 respondents, 4 did not report employee role and were excluded from analysis.  ^b^. Providers included doctors, physician assistants, and nurse practitioners.  ^c.^ Other staff included nursing assistants, administrators, licensed practical nurses, MRI technologists, PhD students, respiratory therapists, clinical research assistants, couriers, imaging technologists, medical students, nuclear medicine technologists, phlebotomists, program assistants, research managers, respiratory therapists, technical editors, and technologists. | | | |
